# Megalourethra with Y-Type Duplication of Urethra Presented as Perianal Fistula: A Rare Case Report

**DOI:** 10.1155/2015/386131

**Published:** 2015-06-03

**Authors:** Shashi Verma, Goto Gangkak, Sher Singh Yadav, Vinay Tomar

**Affiliations:** SMS Medical College & Hospitals, Jaipur 302004, India

## Abstract

Megalourethra with Y-type duplication is an extremely rare anomaly. We report here one such case, diagnosed with retrograde urethrogram, which was done from both penile meatus and perianal opening simultaneously. Patient was successfully treated by laser optical internal urethrotomy (OIU), excision of duplicated urethra, and reduction urethroplasty in a single stage.

## 1. Introduction

Y-type of urethral duplication is a rare congenital anomaly and its association with megalourethra is an extremely rare finding. Y-type of urethral duplication can be considered as a congenital urethroperineal fistula provided ventral urethra is hypoplastic [1]. But all urethroperineal fistulas, which are congenital, should be considered as urethral duplication [2]. Megalourethra is in fact a nonobstructive dilation of urethra, because till now no true anatomic obstruction has been identified. It is of two types, milder and severe types. In milder type, only corpus spongiosum and urethra are involved whereas in severe type both corpus cavernosum and corpus spongiosum are also involved.

## 2. Case Report

**Figure 1:**
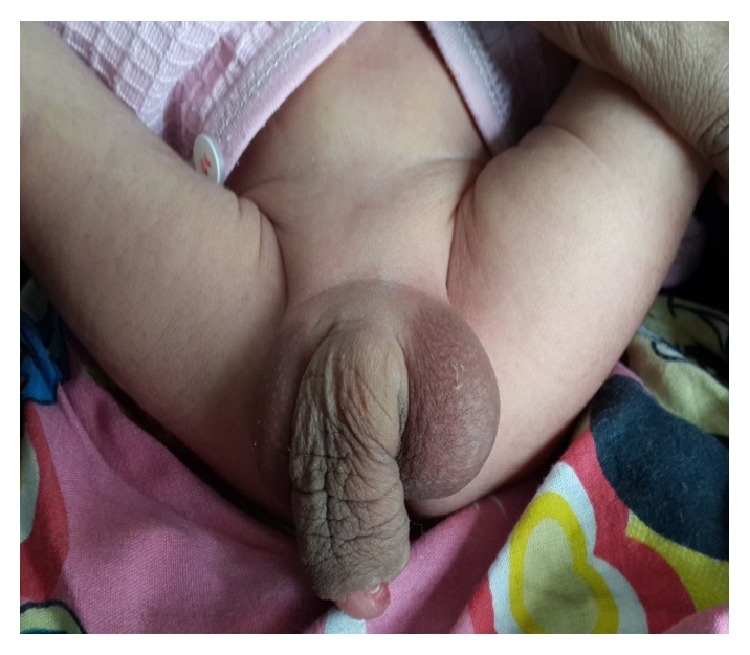
Deformed phallus.

A six-month-old male baby was brought to our department due to deformed phallus (Figure 1). The physical examination revealed enlarged, wide, and soft penis with absence of corpus spongiosum. During voiding, there was dribbling of urine from penile meatus and thin stream of urine was coming from the perianal opening. Beside this, there was a bulge on ventral aspect of penis. Testes were normally descended and overlying skin and musculature of abdominal wall were normal. Ultrasonography showed bilateral hydroureteronephrosis with distended, thick walled bladder. However, renal function was normal.

**Figure 2:**
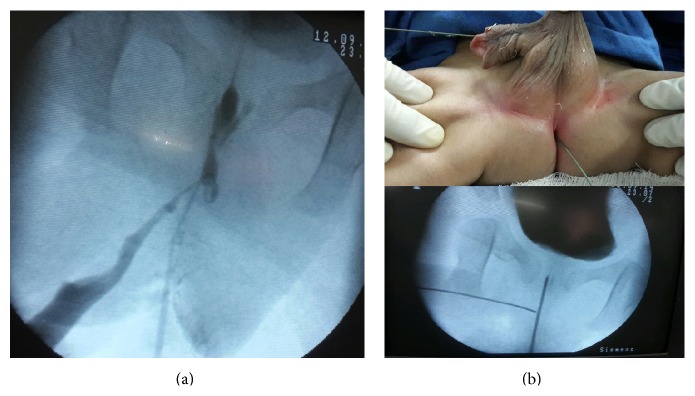
(a) Retrograde urethrography showing Y-type duplication and dilated penile urethra with narrowing in bulbar urethra. (b) Guide wires negotiated through penile meatus and perianal opening.

Retrograde urethrography was done simultaneously from penile meatus and perianal opening which showed Y-type duplication of urethra. It also revealed dilated penile urethra and narrowing in bulbar urethra. Ventral urethra was of narrow caliber throughout its course. There was no vesicoureteral reflux on voiding cystourethrography (Figure 2(a)).

**Figure 3:**
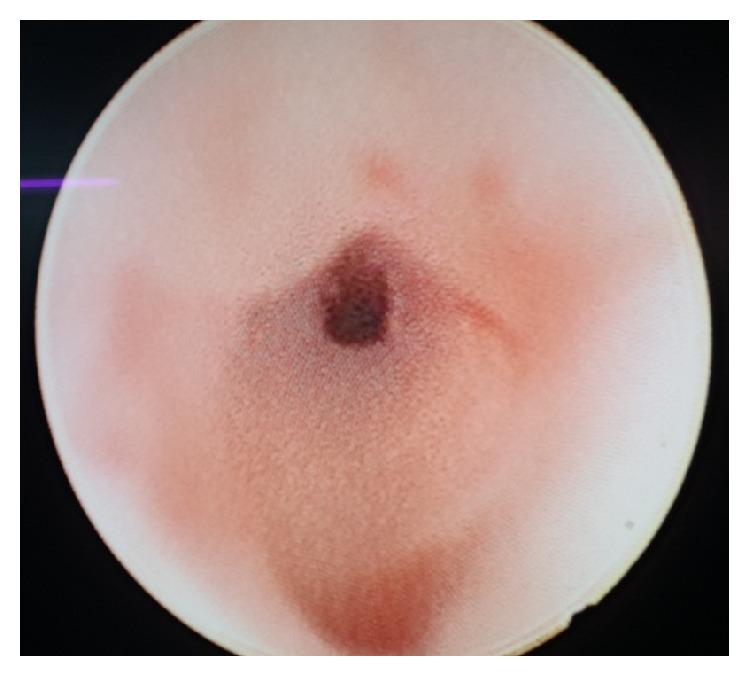
Urethroscopy showed narrowing in distal bulbar urethra.

Guide wires negotiated through penile meatus and perianal opening. Both were intersecting each other in distal bulbar urethra (Figure 2(b)). A urethroscopy was done to confirm the narrowing in distal bulbar urethra (Figure 3).

## 3. Methods

We passed a 0.25′′ guide wire through the narrowing of bulbar urethra with the help of endoscope. Distal bulbar narrowing was dealt with via laser OIU. Duplicated urethra was dissected and divided over a guide wire through the perineal incision. Dilatation of penile urethra was treated by tailoring the redundant urethra. Plication of corporal bodies was also done to support the urethra.

## 4. Result

At two-month follow-up, patient is continent and voiding with good stream from penile meatus. Overall, patient is doing well.

## 5. Discussion

According to Effmann's classification of urethral duplication (UD),* Type IA* is incomplete distal UD*, Type IB* is incomplete proximal UD,* Type IIA1 *is two-meatus noncommunicating urethras arising independently from the bladder,* Type IIA2* is second channel arising from the first and exiting independently,* Type IIB* is complete UD bifurcate and is rejoining at one meatus, and* Type III* isUD as a component of caudal duplication [3]. Our case was a Type IIA2 “Y-type” anomaly according to this classification.

There are two variants of Y-type duplication, usual and unusual. Usual form is defined as hypoplastic dorsal and functional ventral urethra whereas unusual form is defined as normal dorsal and hypoplastic ventral urethra [4]. Embryologically, possible mechanisms for Y-type duplication are (a) vascular accidents leading to fistula formation in dorsal margin of urogenital sinus, (b) defect in closure of urorectal membrane, and (c) inappropriate growth of dorsoinferior wall of urogenital sinus [5].

Megalourethra is associated with other congenital malformations such as prune belly syndrome, dysplasia-hypoplasia, hydronephrosis, hydroureter, undescended testes, and vesicoureteric reflux. Anorectal malformations, posterior urethral valves, and urethral duplication have also been reported with megalourethra [6, 7]. Embryologically, megalourethra is due to (a) defective migration, differentiation, and development of mesenchymal tissues of the phallus and (b) delayed canalization of glans urethra which results in transient urethral obstruction [8]. There is no single theory in literature which can explain the association of megalourethra with Y-type duplication of urethra.

Reduction urethroplasty for treatment of megalourethra was described by Rajendran et al. [9]. Excision of accessory urethra is the main surgical treatment in such kind of duplication [1, 2]. Sclerosis or fulguration of accessory urethra can also be tried [10].

To the best of our knowledge, this may be the first case report of megalourethra with IIA2 “Y-type” urethral duplication in which laser OIU, reduction urethroplasty, and excision of accessory urethra were done in a single stage.
